# Do bears hibernate in the woods? Comment on ‘Why bears hibernate? Redefining the scaling energetics of hibernation’

**DOI:** 10.1098/rspb.2022.1396

**Published:** 2022-10-12

**Authors:** Øivind Tøien, Brian M. Barnes, Thomas Ruf

**Affiliations:** ^1^ Institute of Arctic Biology, University of Alaska Fairbanks, Fairbanks, AK, USA; ^2^ Research Institute of Wildlife Ecology, University of Veterinary Medicine, 1210 Vienna, Austria

Nespolo *et al.* [1] provided a recompilation of published data on energy use during mammalian hibernation based on over-winter decreases in body mass, which led them to three main conclusions: (i) daily energy expenditure during hibernation (DEE_HIB_) scales directly or isometrically with body mass, (ii) energy savings during hibernation become zero compared to remaining at basal metabolic rate (BMR) at body masses above 75 kg (and above 155 kg compared to DEE); thus there would be no or little energy savings in a bear-sized hibernator, and (iii) and that the isometric scaling of DEE_HIB_ is due to an inherent per cell minimum metabolism. In our opinion, there are issues with how these data were selected and compiled. The most important problem is that DEE_HIB_ was not compared to empirical BMR data for each species, but rather drawn from general allometric relationships. Use of species-specific measures of BMR changes the body mass at which regression lines cross and thus where no savings from hibernation can be expected from 75 kg to over 2250 kg. Besides, empirical data have demonstrated that hibernating black bears (*Ursus americanus*) (approx. 100 kg) reduce minimum metabolic rate during hibernation to 25% of BMR [2,3]. Thus, bears indeed hibernate in the woods to save energy.

We also suggest that isometric scaling of DEE_HIB,_ shown previously by Heldmaier *et al.* [4], has an alternative or complementary explanation to minimum cellular metabolism: storage of fat and other substrates used as energy sources during hibernation is limited by body volume and scales isometrically with body mass, and thus DEE_HIB_ will also scale near isometrically with body mass. Since mass-specific BMR increases exponentially with decreasing body mass in mammals, energy savings during hibernation will also increase exponentially as body mass decreases, and this is effected by active suppression of metabolism and decreasing body temperature.

In addition, referring to the original article's table S1, relatively few data are available at high body masses, and the data of the larger species that are compared deserve extra attention. Thus, we replaced data from the American badgers (*Taxidea taxus*), which only have sporadic bouts of daily torpor [5], with DEE_HIB_ data derived from weight loss in European badgers (*Meles Meles*) that show a pattern of decreased body temperature resembling that of black bears [6] and are considered to be hibernators. The metabolic rate saving in black bears listed at the bottom of table S1 is quoted incorrectly and should be a 75% saving with respect to minimum metabolic rate [2]. DEE_HIB_ for black bears was derived from weight loss of both pregnant and non-pregnant black bears in mid-hibernation [7] and should be limited to the non-pregnant bears since there is mass transfer from the sow to cubs during lactation, and females maintain euthermic levels of body temperatures during the gestation period [2,8,9], affecting metabolic rate. The DEE_HIB_ from brown bears by Hilderbrand *et al.* [10] also included pregnant females and was determined over a much longer period of time, from before immergence in October until after emergence in mid-April to early June. Thus, these data also included weight loss during less well-defined transitions in and out of hibernation, when the bears possibly were in a non-hibernating state and females were nursing cubs. Also, data on overwinter weight loss suggesting minimal changes in arctic ground squirrels (*Urocitellus parryii*) are incorrect since they included that from reproductive males, which after ending hibernation gain weight by eating cached food while they develop their testes before they emerge in spring and are weighed [11,12]. Finally, there is no rationale for subtracting interbout arousals from hibernation as was indicated in S1 unless the purpose is to assess minimum metabolism, and then minimum metabolic rate data should be used rather than DEE_HIB_. We redefine DEE_HIB_ to not include arousals using data in the DEE_HIB_ *total* column of the original table S1 as the starting point to represent the revised DEE_HIB_.

As shown in figure 1, these changes to the data result in a change in exponent of the logarithmic regression of DEE_HIB_ versus body mass, going from a slightly positive exponent (1.022) to slightly negative exponent (0.981) and a better correlation (*R*^2^ = 0.977 versus 0.965). At a body mass of 75 kg, it contributes to a decrease in DEE_HIB_ of 22%. The main problem in the original article [1], however, is that it uses inaccurate estimates of BMR by use of White & Seymore's [21] allometric equation, which is based on metabolic rate data that have been temperature compensated to a body temperature of 36.2°C, with a Q_10_ of 3.0 (the dotted line in figure 1). This compensation was aimed at comparing a very wide group of mammals over a large range of body masses, but does not represent the actual predicted metabolic rate at normal body temperature. Black bears have a considerably higher body temperature, at 37.8°C, and have a relatively high BMR of 0.276 ml/(g h) [2], and are thus very far off from the allometry based estimate of 0.111 ml/(g h). If empirically measured BMR is used where available instead of allometrically predicted data, the resulting log–log regression for BMR for the included species intersects with DEE_HIB_ at a body mass of 2268 kg, much higher than stated in the original article (figure 1). Speakman & Król's [22] allometric estimates for DEE are also likely too low at higher body mass for the species included as they become less than our BMR estimates at body masses beyond 1517 kg. A doubly labelled water study in polar bears [23] found a DEE that is twice the Speakman & Krol [22] estimate at the average body mass of 182 kg. Comparisons of both BMR and DEE to DEE_HIB_ clearly show that the discussion of the original article of why bears hibernate is not supported: hibernating bears need to decrease DEE_HIB_ well below BMR to make their fat reserves last through hibernation, even when not pregnant and lactating, thus the original statement of the article should not be standing.

**Figure 1.**
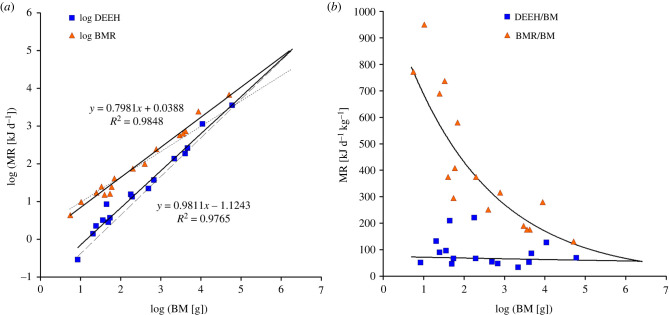
(*a*) Logarithm of metabolic rates (MR; kJ d^−1^) versus logarithm of BM (g). Blue squares: corrected log DEE_HIB_ data as described in the text; orange triangles: log BMR data for the same species derived from appendix 1 in [13] except for *Dromiciops gliroides* [14], U. parryii [15], *Marmota monax* [16], *Tachyglossus aculeatus* [17], *U. americanus* [2], *Marmota marmota* [18], *Hipposideros* *terasensis* [19] and *M. meles* [20]; dotted line is the allometric estimate for log BMR based on White & Seymour [21] used in the original article, dashed line estimate for log DEE_HIB_ of the original article [1]. (*b*) Same data and regression lines expressed as kJ d^−1^ kg^−1^ on linear *y*-axis versus BM (g) expressed on logarithmic *x*-axis, showing the exponential increase in mass specific BMR and need to save energy with decreasing body mass below 2268 kg, while mass-specific DEE_HIB_ remains constant. (Online version in colour.)

## Data Availability

A spreadsheet with the data and sources for figure 1 is available in the Dryad Digital Repository: https://doi.org/10.5061/dryad.msbcc2g1w [24].
